# The relationship between hypovitaminosis D and metabolic syndrome: a cross sectional study among employees of a private university in Lebanon

**DOI:** 10.1186/s40795-018-0243-x

**Published:** 2018-10-11

**Authors:** Rachelle Ghadieh, Jocelyne Mattar Bou Mosleh, Sibelle Al Hayek, Samar Merhi, Jessy El Hayek Fares

**Affiliations:** 1 Department of Nursing and Health Sciences, Notre Dame University- Louaize (NDU), Zouk Mosbeh, Lebanon; 2 Department of Endocrinology, Diabetes, Metabolism and Eating Disorders, University Hospital of Saint-Etienne, Saint-Etienne Cedex, France; 3 Department of Dietetics and Nutrition, The University of Kansas Medical Center, 3901 Rainbow Blvd, Kansas City, KS 66160 USA

**Keywords:** Metabolic syndrome, Vitamin D, Waist circumference, Blood pressure, Triglycerides, Fasting blood glucose

## Abstract

**Background:**

The prevalence of low vitamin D status and metabolic syndrome is increasing globally and in Lebanon. The objectives of this study are to assess the prevalence of metabolic syndrome (MetS) and its components (elevated triglycerides, low HDL, abdominal obesity defined by high waist circumference, hypertension, impaired fasting blood glucose) and investigate the association between serum 25-hydroxyvitamin D (25(OH)D) concentrations and MetS and its components among a sample of Lebanese adults.

**Methods:**

A cross-sectional study was carried out on Notre Dame University employees. A background questionnaire, a short-form of the International Physical Activity Questionnaire and a food frequency questionnaire were administered. Participants were invited to the nutrition laboratory to gather data on anthropometric (height, waist circumference, weight, body composition and body mass index) and biochemical measurements (serum vitamin D, triglycerides, HDL and fasting blood glucose). Vitamin D status was assessed according to the Institute of Medicine cut-offs (inadequate or adequate: 25(OH)D < or ≥ 50 nmol/L).The definition of the Third Report of the National Cholesterol Education Program was used to identify individuals who had MetS. The data were analyzed using the SPSS version 22. *P* < 0.05 was considered statistically significant.

**Results:**

A total of 344 participants (age range of 20 to 74 years) were included in the study. The prevalence of MetS was 23.5%. Among MetS components, central obesity was the most prevalent condition (50.6%), while the least prevalent was impaired fasting blood glucose (20.3%). The odds of having MetS were found to be 2.5 (95% CI 1.3–4.7) higher among those with inadequate vitamin D status, than among those with adequate vitamin D status while controlling for important confounders (age, marital status, education level, income, medical morbidity, smoking and percent body fat and gender). Among the components of MetS, only hypertriglyceridemia (OR: 2.4, 95%CI: 1.3–4.2) and low HDL (OR: 1.8, 95% CI: 1.0–3.0) were associated with inadequate vitamin D status while controlling for important confounders.

**Conclusions:**

Early identification and control of risk factors for cardiovascular diseases in the primary care level is needed, particularly among adults who have low vitamin D status, are obese, and have low income level.

**Electronic supplementary material:**

The online version of this article (10.1186/s40795-018-0243-x) contains supplementary material, which is available to authorized users.

## Background

According to the International Osteoporosis Foundation (IOF), hypovitaminosis D is prevalent in all regions around the world, mostly common in South Asia and the Middle East [1]. There is a growing body of evidence suggesting a genetic basis for low 25 hydroxyvitamin D (25(OH)D) concentrations in the Middle Eastern region. Single nucleotide polymorphism in vitamin D pathway genes has been identified among people of Middle Eastern decent predisposing them to low vitamin D status [2]. Multiple studies have reported a high prevalence of low vitamin D status in Lebanon ranging from 44 to 73% depending on the population and cutoffs used to define low vitamin D status [3–5]. For instance, Hoteit et al., 2014 reported that 60% of Lebanese adults had 25(OH)D <  20 ng/mL [4]. While, Rachkidi and Aoun, 2015 reported a higher prevalence of low vitamin D status among ambulatory patients (73.33%), using a higher cutoff (25(OH)D < 30 ng/mL) [5]. Besides the traditional role of vitamin D in bone metabolism, the literature has recently highlighted important non-traditional roles of this vitamin. An increasing body of evidence is suggesting an association between low concentrations of vitamin D and the metabolic syndrome (MetS), a cluster of conditions that include abdominal obesity, elevated blood pressure (BP), elevated triglycerides (Tg), elevated fasting blood glucose (FBG), and reduced high-density lipoprotein (HDL) cholesterol [6, 7]. This syndrome has been established as a risk factor for type 2 diabetes mellitus and cardiovascular diseases [8, 9].

The International Diabetes Federation (IDF) estimates that one-quarter of the world’s population has MetS [7]. In Lebanon, based on the IDF classification criteria, the overall prevalence of the MetS (two or more factors in addition to abdominal obesity) was 31.2% in a random sample of 499 Lebanese adults across the six administrative districts of Lebanon [10].

Several epidemiologic studies have reported an association between vitamin D deficiency and MetS in different population groups [11–13], however results were not always consistent [14, 15]. The controversial association between vitamin D and MetS could be due to different definitions of MetS, different definitions of healthy vitamin D status, or even the inconsistent use of MetS as a dependent or independent variable.

Even though, the potential mechanism behind the association between low vitamin D status and MetS is not fully elucidated, several pathways have been suggested. Foss hypothesized that in cold weather, in order to survive ‘a ‘winter response’ was generated [16]. These phenotypic metabolic and physiological changes (including hypertension and insulin resistance), observed in MetS, could result from a ‘winter metabolism’ which increases thermogenic capacity. At very low temperatures, survival might depend on additional fuel being available for shivering, and the lipidaemia, glycaemia, and elevated intramuscular Tg observed in MetS to provide supplementary fuel reserves. According to Foss, the stimulus for the ‘winter response’ was low vitamin D concentrations [16]. Further, low vitamin D status can increase the likelihood of MetS since, decreased levels of vitamin D may cause insulin resistance, the underlying link between the different components of MetS [17]. Vitamin D reduces the insulin resistance in the surrounding tissues and thus reduces the excessive insulin release in response to increased blood sugar due to insulin resistance. As a result, it increases the insulin sensitivity. Therefore, vitamin D deficiency is a risk factor for MetS and insulin resistance. Vitamin D does not only increase the production capacity of β-cells, it also accelerates the pro-insulin conversion [18].”

On the other hand, some studies discussed reverse causality, suggesting that obesity, a component of MetS, could lead to low vitamin D status [19]. For instance, larger storage capacity for vitamin D in obese individuals leads to lower circulating 25(OH)D concentrations [20].

Given the high prevalence of MetS and low vitamin D status in the Lebanese population and the limited number of studies assessing this association in the Middle Eastern ethnic groups [3, 21], our main objective was to assess the association between MetS and vitamin D status, after controlling for important confounders. We hypothesized that there is an inverse independent association between vitamin D status and MetS.

## Methods

### Study design and recruitment methods

A cross-sectional study was carried out on Notre Dame University (NDU) employees in the three campuses (Zouk Mosbeh, North, and Shouf). Prior to the initiation of the study, the protocol was approved by the Institutional Review Board of NDU and the study was conducted in accordance with the ethical standards laid down in the 1964 Declaration of Helsinki and its later amendments. In October 2016, an email was sent to all employees of NDU to invite them to join the study. In addition, four nutritionists visited faculty members and staff in their offices to explain the purpose of the study. Assuming that the prevalence rate of MetS in Lebanese adults was 31% [10], the sample size was calculated to be 326 individuals, with a power of 87% using G*Power Version 3.1.3.2 software (Program written, concept and design by Franz, Universitat Kiel, Germany) [22]. Among 600 contacted employees, 360 (60.0%) accepted to participate and were screened for eligibility. Exclusion criteria included pregnancy, lactation, failure to complete the questionnaires and presence of a pacemaker or metal pieces in the participant’s body. Those who were found to be eligible (*n* = 344) were asked to sign an informed consent form and then contacted by the study investigators to arrange for a 30-min face-to-face interview. The interview included a background questionnaire, short-form of the International Physical Activity Questionnaire (IPAQ) and a food frequency questionnaire (FFQ). An identification number was assigned for each participant. All questionnaires were labeled using codes.

### Dietary intake

Vitamin D intake was assessed using a revised version of an existing prototype FFQ that was previously developed by study investigators to evaluate vitamin D intake [23]. The FFQ was revised to include different kinds of products high in vitamin D that are not typically consumed by Canadians, for example different kinds of local fish and cheeses were included. The FFQ was comprised of 9 food items. For each food item, participants were asked to mark their frequency of intake of a designated serving/portion size per day/week/months or rarely/never during the past year. Photos and food models were provided to study participants to assist them in estimating portion size. The FFQ included full-fat/low-fat dairy products, eggs and egg based dishes, fish, margarine, cheeses and ice cream. Estimates of vitamin D intake of each participant were generated using the Nutritionist-Pro-Diet Analysis software version 31.0 (Axxya Systems, Woodinville, WA, USA). Lebanese dishes and recipes were composed and entered using this software according to the Middle-East Food Composition Tables and the Canadian Nutrient File [24].

### Socio-demographic and lifestyle variables

The background questionnaire included questions on socio-demographic and, lifestyle characteristics. Examples of questions included: (i) socio-demographic factors: age, marital status, and level of education. (ii) lifestyle factors: alcohol intake (never/ occasionally, 1–2 drinks per day, 1–2 drinks per week, more than 2 drinks per day), smoking (smoker/non smoker) and sunscreen use (usage of products applied to skin that help prevent the sun’s ultraviolet (UV) radiation from reaching the skin).

### Physical activity levels

The IPAQ-Short Form, a 7-item self-administered questionnaire, tested for use in assessing physical activity (PA) among adults, was used to assess PA level of the study participants [24]. IPAQ asks about three specific types of activities: walking, moderate and vigorous physical activities and time spent by an individual [25]. The items were structured to provide separate scores on each of these activities. Using the following values, Walking = 3.3 METs, Moderate PA = 4.0 METs and Vigorous PA = 8.0 METs, four continuous scores were calculated. These scores were then added to calculate the total physical activity score. Low-level, moderate-level and high-level PA were defined by scores of less than 600 MET-minutes per week, between 600 to less than 3000 MET-minutes per week, and of 3000 or more MET-minutes per week, respectively.

### Clinical assessment

Study participants were asked to pass by the Nutrition Laboratory of each campus for anthropometric assessment, BP measurement and a blood draw, after an overnight fast (NPO after 12 midnight). Nutritionists measured height and waist circumference (WC). Height was measured to the nearest 0.1 cm according to the following protocol: no shoes, heels together and head touching the stadiometer’s ruler aligned horizontally. For WC measurement, a non-stretchable tailor measuring tape was placed around the bare abdomen just above the hip bone and parallel to the floor. Participants were asked to exhale, and measurement was taken to the nearest centimeter at the midpoint between the bottom of the rib cage and above the top of the iliac crest during minimal respiration [26]. WC values were classified as risky using the World Health Organization (WHO) cutoffs for men > 102 cm and women > 88 cm [27]. Weight and body composition were measured using the bioelectrical impedance analysis (BIA) machine InBody 720 (Biospace, Seoul, Korea). BIA is widely used in research since it is quick, safe, and inexpensive [28]. By impedance, BIA measures body water, then estimates fat mass and fat-free mass [29]. The BIA machine was transported to different campuses for data collection and it was calibrated prior to its use. Participants were asked to arrive on an empty bladder and stomach. Before each trial, subjects were asked to wipe the palm of their hands and soles using a specific Biospace Electrolyte tissue to enhance electrical conductivity. The participants were asked to stand on the machine barefooted, without wearing any metal/jewelry. Body Mass Index (BMI) was calculated based on the measured weight and height as: Weight (kg)/ Height (m^2^). Underweight was defined as BMI < 18.5 kg/m^2^, normal weight: 18.5–24.9 kg/m^2^, overweight: 25–29.9 kg/m^2^, and obese ≥30 kg/m^2^ [27].

BP was measured according to the National High Blood Pressure Education Program’s guidance on optimal BP measurement techniques [30]. Each patient was seated comfortably, with back supported, legs uncrossed, and upper arm bared. Patient’s arm was supported at heart level. Cuff bladder encircled 80% or more of the patient’s arm circumference. Mercury column was deflated at 2 to 3 mm per second. The first and fifth audible Korotkoff sounds were recorded as systolic blood pressure (SBP) and diastolic blood pressure (DBP), respectively. Three BP measurements were obtained at an interval of ten minutes each, the measurements were given to the nearest 2 mmHg and the mean was calculated.

A nurse collected a fasting sample of blood for assessment of 25(OH)D, Tg, HDL and FBG. Samples collected at the regional campuses were transported to the Zouk Mosbeh campus on ice on daily basis. They were stored at -20 °C for a maximum period of 6 weeks before analysis.

Serum 25(OH)D concentrations were measured using Enzyme Linked Immune Sorbent (ELISA) technique using the Calbiotech 25(OHD) ELISA Kit (Spring Valley, California, USA), with an intra-assay coefficient of variation of 4.95% and an inter-assay coefficient of variation of 5.63%, and a sensitivity of 0.67 ng/mL, at the Biology laboratory in the Zouk Mosbeh campus. Vitamin D status was assessed according to the the Institute of Medicine cut-offs (inadequate or adequate: 25(OH)D < or ≥ 50 nmol/L /< or ≥20 ng/mL) [1, 31].

Serum Tg, HDL-cholesteroland FBG were measured using a dry chemistry analyzer Vitros 250 (Ortho Clinical Diagnostic, Raritan, New Jersey, USA) available at the same laboratory. The definition of the Third Report of the National Cholesterol Education Program (NCEP-ATP III) was used to identify individuals who had MetS. Participants who met three or more of the following criteria were considered as having MetS: WC ≥102 cm in men or ≥ 88 cm in women, serum Tg level ≥ 150 mg/dL, HDL-cholesterol levels< 40 mg/dL in men or < 50 mg/ dL in women, impaired FBG ≥100 mg/dL or on antidiabetic treatment and BP ≥130/ 85 mmHg or on treatment for hypertension [32].

### Variables of the study

The variables included in the study were as follow: age, marital status, educational level, income, medical morbidity, intake of vitamin D and supplements in the last three months, daily exposure to direct sunlight, use of sunscreen, smoking, alcohol drinking, physical activity level, vitamin D intake, vitamin D concentration, BMI, percent body fat, WC, MetS status, BP, FBG, Tg levels and HDL.

### Pilot testing

The questionnaires were pre-tested during the month of September 2016. The draft questionnaires were tried out on a random sample of 30 NDU staff and faculty members (14% of the calculated sample size). Pilot testing was performed to measure how much time it takes to complete each questionnaire, and clarify question wording, or response categories when necessary and then questionnaires were revised as needed prior to the launching of the study. This sample was not included in data analyses.

### Statistical analysis

Quantitative and qualitative measurements were summarized as mean ± standard deviation and n (%), respectively. Participant characteristics were compared according to MetS status and vitamin D status using chi-square/Fisher’s Exact tests for categorical variables and Independent two-sample t−/Mann-Whitney U tests for continuous variables. Binary logistic regression was used to examine the association between vitamin D status (using IOM cutoffs) and MetS controlling for important confounders having a *p*-value < 0.25 in the bivariable analysis (age, marital status, education level, income, medical morbidity, smoking and percent body fat) in addition to gender. Binary logistic regression was used to examine the association between vitamin D status (using IOM cutoffs) and MetS components (Hypertriglyceridemia, low HDL levels, risky WC, hypertension and impaired fasting glucose) controlling for important confounders having a p-value < 0.25 in the bivariable analysis (age, marital status, education level, income, medical morbidity, smoking and percent body fat) in addition to gender. The data were analyzed using the Statistical Package for Social Sciences software (SPSS) version 22. A *P*-value of less than 0.05 was considered statistically significant.

## Results

### Characteristics of the respondents

Sample characteristics (socio-demographic, anthropometric, lifestyle and biochemical factors) of the study population were shown in Table 1. A total of 344 participants (50% men and 50% women) were included in the analyses with an age range of 20 to 74 years, 78.2% of participants held a bachelor degree or above. Among participants, 61.6% were non-smokers and 70.9% did not use sunscreen.

**Table 1:** Sample characteristics (socio-demographic, lifestyle, anthropometric, dietary intake and biochemical factors) in employees of a private Lebanese university

Characteristic	Total (*n* = 344)		Men (*n* = 172)		Women (*n* = 172)		
	n or mean	% or SD	n or mean	% or SD	n or mean	% or SD	Significance
Age (years)	42.6	11.5	45.6	12.1	39.5	10.2	0.000
Marital status							
Single/ Separated/ Divorced	119	34.6	53	30.8	66	38.4	0.174
Married	225	65.4	119	69.2	106	61.6	
Education level							
High school/BT^1^	75	21.8	48	27.9 ^a^	27	15.7 ^a^	0.013
Bachelor degree	87	25.3	36	20.9 ^b^	51	29.6 ^b^	
Graduate	182	52.9	88	51.2 ^a,b^	94	54.7 ^a,b^	
Income ($)							
< 2250	112	32.6	61	35.5 ^a^	51	29.6 ^a^	0.033
2250–4000	87	25.3	33	19.2 ^a^	54	31.4 ^b^	
> 4000	145	42.2	78	45.3 ^a^	67	39.0 ^a^	
Medical morbidity							
No	202	58.9	91	53.2	111	64.5	0.043
Yes	141	41.1	80	46.8	61	35.5	
Intake of vitamin D supplement, past 3 months							
No	270	78.5	142	82.6	128	74.4	0.088
Yes	74	21.5	30	17.4	44	25.6	
Intake of other supplement, past 3 months							
No	252	73.3	133	77.3	119	69.2	0.113
Yes	92	26.7	39	22.7	53	30.8	
Daily exposure to direct sunlight							0.008
≤ 15 mins	136	39.5	66	38.4 ^a^	70	40.7 ^a^	
16–60 min	112	32.6	46	26.7 ^a^	66	38.4 ^b^	
> 60 mins	96	27.9	60	34.9 ^a^	36	20.9 ^b^	
Use sunscreen							
No	244	70.9	165	95.9	79	45.9	0.000
Yes	100	29.1	7	4.1	93	54.1	
Smoking							
No	212	61.6	93	54.1	119	69.2	0.006
Yes	132	38.4	79	45.9	53	30.8	
Alcohol drinking							
No	255	74.1	112	65.1	143	83.1	0.000
Yes	89	25.9	60	34.9	29	16.9	
Physical activity level							
Low	221	64.2	10.3	59.9	118	68.6	0.115
Moderate/High	123	35.8	69.0	40.1	54.0	31.4	
Vitamin D intake (μg)	2.24	3.18	2.46	4.0	2.03	2.04	0.209
Vitamin D concentration (ng/mL)	28.2	13.9	28.4	15.0	28.1	12.8	0.846
BMI (kg/m^2^)							
Underweight	2	0.6	0	0.0 ^a,b^	2	1.2 ^a,b^	0.000
Normal	122	35.5	27	15.7 ^a^	95	55.2 ^b^	
Overweight	130	37.8	86	50.0 ^a^	44	25.6 ^b^	
Obese	90	26.2	59	34.3 ^a^	31	18.0 ^b^	
Percent body fat (%)	30.90	7.899	27.796	6.930	33.973	7.608	0.000
Waist circumference (cm)	96.5	12.2	102.1	10.5	90.9	11.1	0.000
MetS^2^ status							
No	263	76.5	113	65.7	150	87.2	0.000
Yes	81	23.5	59	34.3	22	12.8	
Waist circumference risky							
No^3^	170	49.4	85	49.4	85	49.4	1
Yes	174	50.6	87	50.6	87	50.6	
Blood pressure							
Healthy	271	78.8	116	67.4	155	90.1	0.000
Unhealthy^4^	73	21.2	56	32.6	17	9.9	
Fasting blood glucose							
Normal	274	79.7	119	69.2	155	90.1	0.000
Impaired^5^	70	20.3	53	30.8	17	9.9	
TG levels							
Normal TG levels	232	67.4	88	51.1	144	83.7	0.000
Hypertriglyceridemia^6^	112	32.6	84	48.8	28	16.3	
HDL							
Normal	262	76.2	129	75	133	77.3	0.704
Low^7^	82	23.8	43	25	39	22.7	

^1^Baccalaureat Technique

^2^Metabolic Syndrome defined according the NCEP-ATP III [63]

^3^WC < 88 cm for women and < 102 cm for men [27]

^4^blood pressure ≥ 130/ 85 mmHg [63]

^5^fasting blood glucose ≥100 mg/Dl [63]

^6^triglycerides level ≥ 150 mg/dL [63]

^7^HDL cholesterol levels< 40 mg/dL in men or < 50 mg/ dL in women [63]

Columns with superscripts without a common symbol differ, *P* < 0.05

### Prevalence of MetS

The prevalence of MetS was 23.5% in the overall study population. Among MetS components, central obesity was the most prevalent condition (50.6%), while the least prevalent condition was impaired FBG (20.3%). The prevalence of other MetS components were as follows 21.2% for elevated BP, 23.8% for low HDL and 32.6% for hypertriglyceridemia. Means of individual MetS components were 138.0 ± 81.9 mg/dL (Tg), 94.7 ± 25.8 mg/dL (FBG), 53.6 ± 14.2 mg/dL (HDL) and 96.5 ± 12.15 cm (WC) (data not shown). Men had a higher prevalence of MetS (34.3%) compared to women (12.8%) (*p* < 0.001). (Table 1).

### Vitamin D status

The mean 25(OH)D concentration was 28.2 ± 13.9 ng/ml and the mean dietary vitamin D intake was 2.24 ± 3.18 μg (Table 1). Neither vitamin D concentration nor intake differed by gender.

### Association between socio-demographic, anthropometric and lifestyle factors and MetS

The associations of socio-demographic, anthropometric and lifestyle factors with MetS were shown in Table 2. Among both genders, individuals with MetS had an older age compared to individuals who didn’t have MetS (*P* = 0.000). Among individuals with MetS, 18.7% had a graduate degree while 38.7% had a high school degree (*p* = 0.002). Among individuals with MetS, 34.8% had a monthly income < 2250 $ while 20.7% had a monthly income > 4000$ (*p* = 0.001). Further analyses have shown that a higher prevalence of smoking and obesity was observed among individuals who have the lowest education and monthly income level (*p* value< 0.05) (data not shown). Also, 43.0% of smokers had MetS compared to 26.9% of nonsmokers (*p* = 0.039). Among men, the mean age of individuals was higher among those with MetS compared to those who did not have MetS (49.9 vs 43.3, *p* = 0.000). Among men, education level, income, smoking and BMI were associated with MetS. Among women, age and BMI were associated with MetS.

**Table 2:** Association of socio-demographic, anthropometric and lifestyle factors with metabolic syndrome in employees of a private Lebanese university

Characteristic	Total (*n* = 344)			Men (*n* = 172)			Women (*n* = 172)		
	No MetS	MetS^1^	*P* value	No MetS	MetS	*P* value	No MetS	MetS	*P* value
	mean ± SD or n(%)			mean ± SD or n(%)			mean ± SD or n(%)		
Age (years)	40.6 ± 11.3	49.0 ± 9.7	0.000	43.3 ± 12.5	49.9 ± 10.0	0.000	38.5 ± 10.0	46.7 ± 8.6	0.000
Marital status			0.227			0.813			0.362
Single/ Separated/ Divorced	96 (80.7%)	23 (19.3%)		36 (67.9%)	17 (32.1%)		60 (90.9%)	6 (9.1%)	
Married	167 (74.2%)	58 (25.8%)		77 (64.7%)	42 (35.3%)		90 (84.9%)	16 (15.1%)	
Education level			0.002			0.023			0.143
High school / BT^2^	46 (61.3%) ^a^	29 (38.7%) ^a^		24 (50.0%) ^a^	24 (50.0%) ^a^		22 (81.5%)	5 (18.5%)	
Bachelor degree	69 (79.3%) ^b^	18 (20.7%) ^b^		27 (75.0%) ^b^	9 (25.0%) ^b^		42 (82.4%)	9 (17.6%)	
Graduate	148(81.3%) ^b^	34 (18.7%) ^b^		62 (70.5%) ^b^	26 (29.5%) ^b^		86 (91.5%)	8 (8.5%)	
Income ($)			0.001			0.023			0.168
< 2250	73 (65.2%) ^a^	39 (34.8%) ^a^		32 (52.5%) ^a^	29 (47.5%) ^a^		41 (80.4%)	10 (19.6%)	
2250–4000	75 (86.2%) ^b^	12 (13.8%) ^b^		25 (75.8%) ^b^	8 (24.2%) ^b^		50 (92.6%)	4 (7.4%)	
> 4000	115 (79.3%) ^b^	30 (20.7%) ^b^		56 (71.8%) ^b^	22 (28.2%) ^b^		59 (88.1%)	8 (11.9%)	
Medical morbidity			0.000			0.004			0.000
No	174 (86.1%)	28 (13.9%)		69 (75.8%)	22 (24.2%)		105 (94.6%)	6 (5.4%)	
Yes	88 (62.4%)	53 (37.6%)		43 (53.8%)	37 (46.2%)		45 (73.8%)	16 (26.2%)	
Smoking			0.006			0.039			0.622
No	173 (81.6%)	39 (18.4%)		68 (73.1%)	25 (26.9%)		105 (88.2%)	14 (11.8%)	
Yes	90 (68.2%)	42 (31.8%)		45 (57.0%)	34 (43.0%)		45 (84.9%)	8 (15.1%)	
Alcohol drinking			0.654			1			0.377
No	197 (77.3%)	58 (22.7%)		74 (66.1%)	38 (33.9%)		123 (86.0%)	20 (14.0%)	
Yes	66 (74.2%)	23 (25.8%)		39 (65.0%)	21 (35.0%)		27 (93.1%)	2 (6.9%)	
Physical activity level			0.698			0.477			0.841
Low	167 (75.6%)	54 (24.4%)		65 (63.1%)	38 (36.9%)		102 (86.4%)	16 (13.6%)	
Moderate/High	96 (78.0%)	27 (22.0%)		48 (69.6%)	21 (30.4%)		48 (88.9%)	6 (11.1%)	
BMI (kg/m^2^)			0.000			0.000			0.000
Underweight	2 (100.0%) ^a,b,c^	0 (0.0%) ^a,b,c^		–	–		2 (100.0%) ^a,b,c^	0 (0.0%) ^a,b,c^	
Normal	122 (100.0%) ^c^	0 (0.0%) ^c^		27 (100%) ^a^	0 (0.0%) ^a^		95 (100.0%) ^c^	0 (0.0%) ^c^	
Overweight	101 (77.7%) ^b^	29 (22.3%) ^b^		63 (73.3%) ^b^	23 (26.7%) ^b^		38 (86.4%) ^b^	6 (13.6%) ^b^	
Obese	38 (42.2%) ^a^	52 (57.8%) ^a^		23 (39.0%) ^c^	36 (61.0%) ^c^		15 (48.4%) ^a^	16 (51.6%) ^a^	

^1^Metabolic Syndrome defined according the NCEP-ATP III [63]

^2^Baccalaureat Technique

Columns with superscripts without a common symbol differ, *P* < 0.05

### Association between vitamin D status and MetS

The association between vitamin D status and MetS were presented in Table 3. Using the IOM cuttoffs, among all participants, 52.3% of those with inadequate vitamin D status had MetS compared to 47.7% of those with adequate vitamin D status (*p* = 0.010). Among MetS components, only hypertriglyceridemia was associated with inadequate vitamin D status. Only 32.6% of those with inadequate vitamin D status had normal Tg levels compared to 67.4% of those with adequate vitamin D status (*p* = 0.014). In men, neither MetS nor its components were associated with vitamin D status. In women, MetS was associated with vitamin D status.

**Table 3:** Associations between vitamin D status and the metabolic syndrome in employees of a private Lebanese university

Vitamin D Status as defined by the Institute of Medicine (Inadequate: < 20 ng/mL; Adequate: ≥ 20 ng/mL)									
	Total (*n* = 268)^a^			Men (*n* = 142)			Women (*n* = 126)		
Vitamin D Status	Adequate	Inadequate	*P* value	Adequate	Inadequate	*P* value	Adequate	Inadequate	*P* value
	mean ± SD or n(%)			mean ± SD or n(%)			mean ± SD or n(%)		
MetS^1^ status			0.010			0.174			0.021
No	135 (66.5%)	68 (33.5%)		60 (65.2%)	32 (34.8%)		75 (67.6%)	36 (32.4%)	
Yes	31 (47.7%)	34 (52.3%)		26 (52.0)	24 (48.0%)		5 (33.3%)	10 (66.7%)	
Waist circumference risky (cm)			0.139			0.606			0.153
No^2^	90 (66.7%)	45 (33.3%)		45 (63.4%)	26 (36.6%)		45 (70.3%)	19 (29.7%)	
Yes	76 (57.1%)	57 (42.9%)		41 (57.7%)	30 (42.3%)		35 (56.5%)	27 (43.5%)	
Blood pressure (mmHg)			0.269			0.949			0.097
Healthy	133 (63.9%)	75 (36.1%)		57 (61.3%)	36 (38.7%)		76 (66.1%)	39 (33.9%)	
Unhealthy^3^	33 (55.0%)	27 (45.0%)		29 (59.2%)	20 (40.8%)		4 (36.4%)	7 (63.6%)	
Fasting blood glucose (mg/dL)			0.675			0.725			1
Normal	135 (62.8%)	80 (37.2%)		62 (62.0%)	38 (38.0%)		73 (63.5%)	42 (36.5%)	
Impaired^4^	31 (58.5%)	22 (41.5%)		24 (57.1%)	18 (42.9%)		7 (63.6%)	4 (36.4%)	
TG levels (mg/dL)			0.014			0.093			0.078
Normal TG levels	120 (67.4%)	58 (32.6%)		49 (68.1%)	23 (31.9%)		71 (67.0%)	35 (33.0%)	
Hypertriglyceridemia^5^	46 (51.1%)	44 (48.9%)		37 (52.9%)	33 (47.1%)		9 (45.0%)	11 (55.0%)	
HDL (mg/dL)			0.146			0.782			0.074
Normal	130 (64.7%)	71 (35.3%)		63 (61.8%)	39 (38.2%)		67 (67.7%)	32 (32.3%)	
Low^6^	36 (53.7%)	31 (46.3%)		23(57.5%)	17 (42.5%)		13 (48.1%)	14 (51.9%)	

^a^Sample excluded participants taking vitamin D supplements (*n* = 76)

^1^Metabolic Syndrome defined according the NCEP-ATP III [63]

^2^WC < 88 cm for women or < 102 cm for men [27]

^3^blood pressure ≥ 130/ 85 mmHg [63]

^4^fasting blood glucose ≥100 mg/dL [63]

^5^triglycerides level ≥ 150 mg/dL [63]

^6^HDL cholesterol levels< 40 mg/dL in men or < 50 mg/ dL in women [63]

Using the IOM cutoff points, the odds of having MetS were found to be approximately 2.5 higher among those with inadequate vitamin D status than among those with adequate vitamin D status while controlling for age, gender, marital status, education level, income, medical morbidity, smoking and percent body fat, however only age, annual income, gender and body composition remained in the model and showed to be statistically significant (Table 4). The odds of having hypertriglyceridemia were approximately 2.4 times higher among those with inadequate vitamin D status compared to those with adequate status after controlling for important confounders. The odds of having low HDL were approximately 1.8 times higher among those with inadequate vitamin D status compared to those with adequate status after controlling for important confounders (Table 5). The other components of MetS (impaired fasting glucose, risky WC and hypertension) were not associated with vitamin D status (Additional file 1: Table S5).

**Table 4:** Logistic regression for metabolic syndrome and vitamin D status using the Institute of Medicine^a^ Cutoffs among employees in a private Lebanese university

	Odds Ratio (OR)	95% C.I		*P* value
		Lower	Upper	
Vitamin D status	2.496	1.315	4.736	0.005
Age, years	1.052	1.022	1.083	0.001
Annual income	0.756	0.591	0.969	0.027
Gender	0.080	0.033	0.189	< 0.001
Body fat percentage	1.180	1.118	1.245	< 0.001

^a^Vitamin D status defined as Adequate: 25hydroxyvitamin D ≥ 20 ng/mL and inadequate: 25 hydroxyvitamin D < 20 ng/mL

Model statistics: R2 = 42.5%, Omnibus test *p* < 0.001, Hosmer Lemeshow test *p* = 0.264

**Table 5:** Logistic regression for components of MetS (hypertriglyceridemia and low HDL) and vitamin D status using the Institute of Medicine^a^ cutoffs among employees in a private Lebanese university

	Odds Ratio (OR)	95% C.I		*P* value
		Lower	Upper	
Hypertriglyceridemia^1^				
Vitamin D status	2.383	1.357	4.184	.002
BMI kg/m^2^	2.683	1.843	3.908	.000
Age, years	1.046	1.021	1.072	.000
Gender	.358	.205	.625	.000
Low HDL^2^				
Education	.691	.507	.942	.019
Smoking	2.117	1.256	3.571	.005
Vitamin D status	1.783	1.050	3.029	.032

^a^Vitamin D status defined as Adequate: 25hydroxyvitamin D ≥ 20 ng/mL and inadequate: 25 hydroxyvitamin D < 20 ng/mL

^1^triglycerides level ≥ 150 mg/dL [63]

Model statistics: R2 = 36.3%, Omnibus test *p* < 0.001, Hosmer Lemeshow test *p* = 0.156

^2^HDL cholesterol levels< 40 mg/dL in men or < 50 mg/ dL in women [63]

Model statistics: R2 = 8.7%, Omnibus test *p* < 0.001, Hosmer Lemeshow test *p* = 0.717

## Discussion

This study found that 23.5% of Lebanese adults in a private university had MetS. All the components of MetS, except for risky WC and low HDL, were more common in men than women. The factors that were associated with MetS included low vitamin D status, older age, low income and high body fat. The odds of having MetS were approximately 2.5 higher among those with inadequate vitamin D status compared to those with adequate status after controlling for important confounders.

The prevalence of MetS (23.5%) reported in the current study was similar to that reported by another study among a national sample of Lebanese adults of 25.4% [33]. Our study findings indicated a prevalence of MetS comparable to that reported in the US of 23.7% [11] and in line with rates reported in the Middle East such as in Oman (21.0%) [34], Turkey (33.4%) [35] and Iran (33.7%) [36]. Similar to Sibai et al., 2008, our study reported a higher prevalence of MetS in men compared to women. In contrast, Erem et al., 2008 reported a higher prevalence of MetS among Turkish women compared to men and Delavari et al., 2009 reported a similar trend among Iranian adults [37, 38]. These gender differences could be attributed to different obesity rates between men and women. For instance, in our study, higher prevalence of overweight and obesity was observed among Lebanese men compared to women and also in the general Lebanese population [39], while in Turkey and in Iran more women were overweight and obese compared to men [10]. It is well-known in the literature that obesity is an important component for MetS [40, 41]. Our results support this assumption as the prevalence of MetS was significantly higher among participants who were overweight or obese compared to normal weight participants. The prevalence of MetS increased across increasing BMI categories, which is coherent with the literature [42]. As BMI and adiposity increase, the adipocytes secrete pro-inflammatory cytokines and chemokines which enhance the inflamed state of the tissues, increase insulin resistance and hence increase the risk of MetS [42]. Further, the concentration of HDL-cholesterol is adversely altered in obesity, with HDL-cholesterol levels associated with both the degree and distribution of obesity [43].

The most prevalent component of MetS was risky WC in both men and women. These results are concordant with the literature in Lebanon and in neighboring countries [10, 33], as obesity has been on the rise in the Middle Eastern region across all age and gender groups [10].

In bivariable and multivariable analyses, our findings showed that age was positively correlated with MetS among both genders, which is in line with previous studies [3, 44, 45]. Several population studies have reported an increase in the prevalence of MetS with age regardless of the definition used [11, 46–48]. It can be inferred that, with age, blood vessels gradually lose elasticity and gain resistance, slowing blood flow. Moreover, with poor circulation, fat is prone to accumulate in the abdomen and release free fatty acids into the serum, leading to higher insulin resistance, elevated serum Tg levels, increased low density lipoprotein- cholesterol (LDL-C) levels, and, consequently, a greater risk of MetS [45].

The prevalence of MetS was inversely associated with income and education, which is in line with the literature in different ethnic and gender groups [49–51]. It is well known in the literature that education and income are strong predictors of health since they may affect lifestyle behaviors and accessibility to healthcare services [51, 52]. This observation was also supported by our results since further data analyses showed that among individuals with low income and education, the prevalence of overweight and obesity and the prevalence of smoking were higher compared to individuals with higher income and education, which would likely increase the risk of MetS. Further, other studies have reported that individuals having low socio-economic background tend to select low-cost, energy-dense food and practice less leisure time activities, which would favor the development of insulin resistance, hypertriglyceridemia, and body weight gain resulting in a higher risk of MetS [53].

In the current study, smoking was associated with MetS in all participants and specifically in men. Previous studies have shown conflicting results regarding the influence of smoking on the prevalence of MetS [54, 55]. Some studies have reported that exposure to tobacco smoke in all forms has been associated with MetS [56, 57]. Smoking has been found to play a causal role in the emergence of core components of MetS. Smoking could increase the risk of abdominal obesity, insulin resistance, leptin resistance, low-grade systemic inflammation, endothelial dysfunction, autonomic dysfunction [58], BP and reduce HDL [57].

Our results showed that a higher percentage of those who had MetS had inadequate vitamin D status compared to those who did not have MetS in the total sample and in women only. However, using the multivariable logistic regression, having inadequate vitamin D status increased the odds of having MetS by 2.5 times. Our results are in line with the literature, for instance Ford et al. showed that the odds of having MetS decreased progressively across increasing quintiles of concentration of 25(OH)D [59]. Previous studies showed that lower serum levels of 25(OH)D were associated with a statistically significant increase in the prevalence of the components of MetS, i.e., elevated BP, elevated Tgs, reduced HDL-C and increased WC [12, 58–61]. In the current study, only hypertriglyceridemia and low HDL were associated with inadequate vitamin D status, while hypertension, risky WC and impaired fasting glucose were not associated with vitamin D status. On the other hand, few studies failed to observe the association between vitamin D status and MetS [3, 14, 15]. It is very likely that the studies that did not observe an association between MetS and vitamin D status were conducted on either high or low risk populations. For instance, Rueda et al. 2008 and Hjelmesaeth et al. 2009 examined this association in morbidly obese individuals reporting very high rates of MetS and other co-morbidities (diabetes type II, hypertension and heart disease), while Gannage-Yared et al., 2009 recruited a sample of young Lebanese adults with healthy weights and low prevalence of MetS [3, 14, 15].

The association between vitamin D status and MetS could be explained by the fact that vitamin D receptors are distributed on vascular smooth muscle, endothelium and cardiomyocytes. Activated 1,25-dihydroxyvitamin D suppresses renin gene expression, regulating the growth and proliferation of vascular smooth muscle cells, cardiomyocytes, and inhibiting cytokine release from lymphocytes. Therefore, the absence of vitamin D receptor activation leads to tonic upregulation of the renin-angiotensin system, eventually leading to hypertension and left ventricular hypertrophy and increasing the likelihood of MetS [61]. Furthermore, another potential mechanism explaining the association between MetS and low vitamin D status is through insulin resistance. Insulin resistance is postulated to be the common underlying pathogenic link between the various components of MetS. Decreased levels of vitamin D may cause insulin resistance [17]. Vitamin D reduces insulin resistance in the surrounding tissues and thus reduces the excessive insulin release in response to increased blood sugar due to insulin resistance. As a result, it increases the insulin sensitivity. Therefore, vitamin D deficiency is a risk factor for MetS and insulin resistance. Vitamin D does not only increase the production capacity of β-cells, it also accelerates the pro-insulin conversion [18]. On the other hand, some studies discussed reverse causality, suggesting that obesity, a component of MetS, could lead to low vitamin D status [19]. For instance, larger storage capacity for vitamin D in obese individuals leads to lower circulating 25(OH)D concentrations [20].

The results of this study should be interpreted with the following limitations in mind. First, subjects were drawn from a single private university in Lebanon and, therefore, the inferences drawn from the results are not generalizable to the Lebanese population. More diverse samples collected by a nation-wide survey should be considered in future research. Second, 25(OH)D concentrations were measured by ELISA, which is not the gold standard technique for vitamin D assessment, but, it is the most commonly used technique in research since it’s relatively simple and inexpensive [62]. Third, as a cross-sectional study, we cannot infer causality from our findings. A longitudinal study design is needed to investigate any causal relationships among the factors included in this study. Fourth, dietary analyses revealed that the Middle Eastern food composition tables did not include the vitamin D content of all foods; accordingly the vitamin D content of these foods had to be derived from the Canadian Nutrient file.

Despite these methodological concerns, to our knowledge, this was the first study to assess the association between MetS and vitamin D status in middle-aged Lebanese adults at risk of developing chronic diseases.

## Conclusion

This study found that about one quarter of adults in our study sample had MetS. In order to promote a more efficient control of MetS, early identification and control of risk factors for cardiovascular diseases in the primary care level is needed, particularly among those who have low vitamin D status, have higher body fat percentage, and have low income level. This justifies the requirement to intensify prevention strategies in Lebanese adults, improving their nutritional status through the prevention and treatment of obesity and promotion of healthy eating. Further research should examine the causal association between low vitamin D status and MetS in order to understand the mechanism behind this association.

## Additional file

Additional file 1: **Table S5**. Logistic regression for components of MetS (impaired fasting blood glucose, risky waist circumference and hypertension) and vitamin D status using the Institute of Medicine cutoffs among employees in a private Lebanese university. Additional table for logistic regression for components of MetS (impaired fasting blood glucose, risky waist circumference and hypertension) and vitamin D status. (DOCX 16 kb)

## Abbreviations

25 (OH)D: 25 hydroxyvitamin D
BIA: Bioelectrical Impedance Analysis
BMI: Body Mass Index
BP: Blood Pressure
DBP: Diastolic Blood Pressure
ELISA: Enzyme Linked Immunosorbent Assay
FBG: Fasting Blood Glucose
FFQ: Food Frequency Questionnaire
HDL: High Density Lipoprotein
IDF: International Diabetes Federation
IOF: International Osteoporosis Foundation
IOM: Institute of Medicine
IPAQ: International Physical Activity Questionnaire
LDL-C: Low Density Lipoprotein Cholesterol
MetS: Metabolic Syndrome
NCEP-ATP III: Third Report of the National Cholesterol Education Program
NDU: Notre Dame University
NOF: National Osteoporosis Foundation.
NPO: Nothing per Mouth
PA: Physical Activity
SBP: Systolic Blood Pressure
SPSS: Statistical Package for Social Sciences
Tg: Triglyceride
VDR: Vitamin D Receptors
WC: Waist Circumference
WHO: World Health Organization
1,25(OH)_2_D: 1–25-dihydroxyvitamin D
