# Supplementary material for: The relationship between hypovitaminosis D and metabolic syndrome: a cross sectional study among employees of a private university in Lebanon
Source: BMC Nutr. 2018 Oct 11;4:36. doi: 10.1186/s40795-018-0243-x (PMC7050701; doi:10.1186/s40795-018-0243-x)
Supplement: Supplementary file 1 — Table S5. Logistic regression for components of MetS (impaired fasting blood glucose, risky waist circumference and hypertension) and vitamin D status using the Institute of Medicine cutoffs among employees in a private Lebanese university. Additional table for logistic regression for components of MetS (impaired fasting blood glucose, risky waist circumference and hypertension) and vitamin D status. (DOCX 16 kb) [file 40795_2018_243_MOESM1_ESM.docx]

Additional file 1: Table S5B

Logistic regression for components of MetS (impaired fasting blood glucose, risky waist circumference and hypertension) and vitamin D status using the Institute of Medicine cutoffs among employees in a private Lebanese university

**Impaired fasting blood glucose^3^**

|  | **Odds Ratio (OR)** | **95 % C.I** | | **P value** |
| --- | --- | --- | --- | --- |
|  |  | **Lower** | **Upper** |  |
| Medical morbidity | 2.823 | 1.422 | 5.607 | .003 |
| BMI kg/m^2^ | 2.514 | 1.592 | 3.969 | .000 |
| Age, years | 1.051 | 1.019 | 1.084 | .002 |
| Gender | .421 | .212 | .835 | .013 |
| \| Model statistics: R2= 36%, Omnibus test p<0.001, Hosmer Lemeshow test p=0.426 \| \| --- \| | | | | |

^3^ fasting blood glucose ≥100 mg/Dl [65]

**Risky waist circumference^4^**

|  | **Odds Ratio (OR)** | **95 % C.I** | | **P value** |
| --- | --- | --- | --- | --- |
|  |  | **Lower** | **Upper** |  |
| BMI kg/m^2^ | 20.129 | 10.641 | 38.078 | .000 |
| Age, years | 1.028 | 1.001 | 1.056 | .039 |
| Gender | 11.092 | 4.859 | 25.322 | .000 |

| Model statistics: R2= 57%, Omnibus test p<0.001, Hosmer Lemeshow test p=0.926 |
| --- |

^4^ WC< 88 cm for women and < 102 cm for men [27]

**Hypertension^5^**

|  | **Odds Ratio (OR)** | **95 % C.I** | | **P value** |
| --- | --- | --- | --- | --- |
|  |  | **Lower** | **Upper** |  |
| BMI kg/m^2^ | 3.181 | 2.019 | 5.012 | .000 |
| Age, years | 1.058 | 1.029 | 1.087 | .000 |
| Gender | .380 | .196 | .738 | .004 |

| Model statistics: R2= 34%, Omnibus test p<0.001, Hosmer Lemeshow test p=0.150 |
| --- |

^5^ Hypertension SBP/DBP ≥130/ 85 mm Hg [65]
